# Authors' reply to the discussion of ‘A COVID‐19 Model for Local Authorities of the United Kingdom’ by Mishra et al. in Session 2 of the Royal Statistical Society's Special Topic Meeting on COVID‐19 transmission: 11 June 2021

**DOI:** 10.1111/rssa.12977

**Published:** 2022-12-08

**Authors:** Swapnil Mishra, James A. Scott, Daniel J. Laydon, Harrison Zhu, Neil M. Ferguson, Samir Bhatt, Seth Flaxman, Axel Gandy

**Affiliations:** ^1^ MRC Centre for Global Infectious Disease Analysis, Abdul Latif Jameel Institute for Disease and Emergency Analytics (J‐IDEA) Imperial College London London UK; ^2^ Department of Mathematics Imperial College London London UK

We are very grateful for the interesting and constructive comments about the papers discussed in this meeting. We will address the points pertaining to our paper.

Professor Gibson and Professor Nason stress that our model does not have NPIs or other information as covariates. We agree that ideally, one would like to do this. However, this conflicts with another goal of our model—to provide estimates on a very regular basis. Given the rapid decision‐making and implementation of new measures, that varied substantially across the United Kingdom, often without exact precedent, it would have meant frequent adjustment of the model and collection and verification of, for example, NPIs for almost 400 areas on a daily basis, making it almost an implausible task without substantial time‐commitment.

Professor Gibson also remarks that our paper does not show prior‐predictive checks to validate the model—we have omitted more detailed model checks due to space constraints. Our R package Epidemia has a full suite to do model checks and we have used them to verify and tune our models. We do agree with Professor Gibson that individual‐based models can be more useful than aggregate models. However, constraints around data availability and compute time makes running individual models on daily basis an unattainable task. Our main objective behind the framework was to have something that can be updated on an almost daily basis, so we opted for simplicity.

Professor Nason raises the point that there are instances of our estimates of $R_{t}$ projections are not overlapping with estimates of $R_{t}$ by Teh et al. in the same time period and how health officials should react to this. As Professor Nason, points out, it is not surprising that with models with different assumptions, sometimes conflicting estimates arise. This may actually be an advantage. If it is well understood how models differ this gives a more varied understanding of the epidemic in these places—for example, one model which relies more on spatial correlation would assume that a local outbreak will start spreading whereas another model with less spatial correlation, such as ours, might not predict this. Model uncertainty can be evident from results from different models.

As Professor Nason points out, we could indeed have chosen other models for the reproduction number than a weekly random walk. We experimented with some of these, for example splines, but in the end, we settled on the random walk, as it proved to be more stable than other approaches, and as it does not project any trends in changes in $R_{t}$ forward in time, which could have lead to unrealistic values of $R_{t}$, particularly around change points of the epidemic.

Dr Jewell raises issues around not having spatial relationships encoded among local areas and using a fixed generation distribution throughout the period. We agree that an ideal model should account for spatial dependence in a realistic way. By not incorporating it we gained at least two advantages. First, easier computational tractability, which allowed us to update the model more frequently and thus produce more current updates. Secondly, and maybe more importantly, it allowed a local ‘accountability’—projections were based mainly on developments within that region, and thus the estimates cannot simply be discounted in one area by saying it only depends on the developments in neighbouring regions. During this epidemic, there were instances of local outbreaks or local reductions that did not, or not quickly, affect neighbouring areas. We experimented with mobility data ourselves, but we chose not to use it, as, in our experience, it tended to large swings in the estimates that were not necessarily reflected in the case data later on, and as mobility data has been, at least when we developed the model, only been available with substantial delays. As far as the use of predetermined generation distribution is concerned, we agree a model that can account for changing generation distribution would be ideal. However, this would again require much more computational expensive frameworks, limiting their use for daily updates. Additionally, identifiability of the parameters would be a major concern—for which we would at least require detailed incidence data not just raw daily case counts.

Overall, we believe that our model has been a useful tool to several decision‐makers during the epidemic. To be prepared for possible future epidemics or a future pandemic, we agree with the discussants that it would be beneficial to develop a detailed and adaptable models that would be able to follow the rapidly changing data availability throughout an epidemic and to address the rapidly changing questions that arise (e.g. effect of vaccinations, age‐dependence, variants, …). These models will most likely require advances in computational methods to be helpful for the rapid decisions needed during an epidemic.

